# Supplementary material for: HMG20A Inhibit Adipogenesis by Transcriptional and Epigenetic Regulation of MEF2C Expression
Source: Int J Mol Sci. 2022 Sep 12;23(18):10559. doi: 10.3390/ijms231810559 (PMC9505946; doi:10.3390/ijms231810559)
Supplement: Supplementary file 1 [file ijms-23-10559-s001.zip › ijms-1858082-supplementary/attachment/Supplementary Figure.pdf]

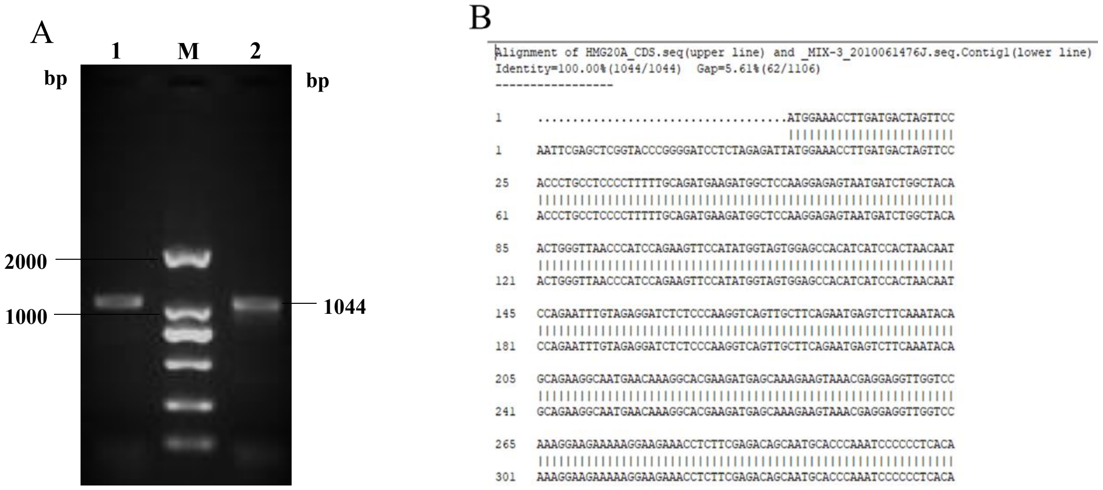

Supplementary figure S1. Cloning of HMG20A gene. (A) The results of PCR amplification. (B) The results of Sanger sequencing.

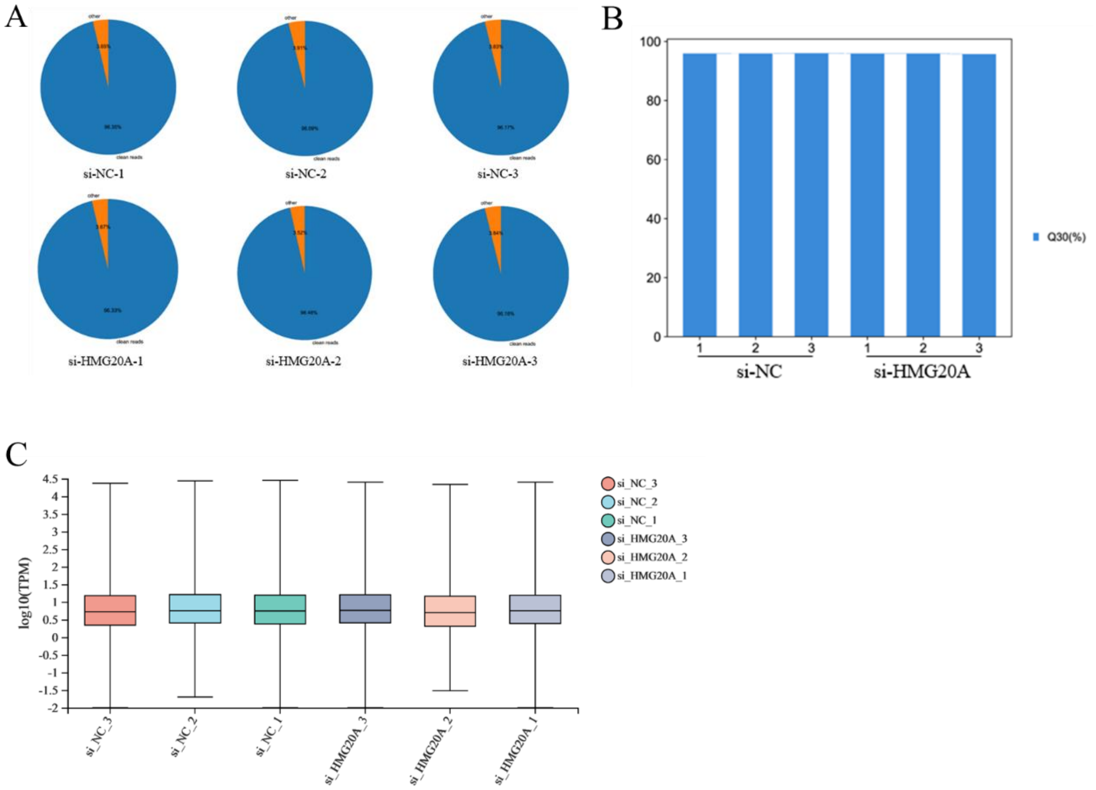

Supplementary figure S2. Evaluation of data quality. (A) Pie chart of the proportion of clean reads to raw reads. (B) Q30 bases rate (%). (C) Sample expression box distribution.
